# Association between tea intake and hospitalized nephrolithiasis in Chinese adults: A case–control study

**DOI:** 10.3389/fnut.2022.1014491

**Published:** 2022-09-28

**Authors:** Yingyu Liu, Shiyuan Bi, Hexiao Li, Jianxiu Shi, Yang Xia, Kaijun Niu, Song Bai

**Affiliations:** ^1^Department of General Surgery, Shengjing Hospital of China Medical University, Shenyang, China; ^2^Department of Obstetrics and Gynecology, Shengjing Hospital of China Medical University, Shenyang, China; ^3^Department of Urology, The First Affiliated Hospital of China Medical University, Shenyang, China; ^4^Department of Clinical Epidemiology, Shengjing Hospital of China Medical University, Shenyang, China; ^5^Nutritional Epidemiology Institute and School of Public Health, Tianjin Medical University, Tianjin, China; ^6^Health Management Centre, Tianjin Medical University General Hospital, Tianjin, China; ^7^Department of Urology, Shengjing Hospital of China Medical University, Shenyang, China

**Keywords:** nephrolithiasis, case control study, tea, diet, risk factors

## Abstract

**Introduction and aim:**

Nephrolithiasis is one of the most common urological disorders worldwide. Tea is one of the most popular drinks worldwide. This study aimed to explore the association between tea intake and hospitalized nephrolithiasis in Chinese adults.

**Methods:**

The patients and healthy participants were from the Shenyang sub-cohort of Tianjin Chronic Low-Grade Systemic Inflammation and Health Cohort Study. After selecting and matching by age (±1 year) and sex using the 1:2 ratio, 834 participants were included in this study. Of these, 278 patients had hospitalized nephrolithiasis and 556 were healthy controls. The tea intake was assessed using a validated self-administered food frequency questionnaire. Multivariate conditional logistic regression analysis was used to evaluate the association between tea intake and hospitalized nephrolithiasis.

**Results:**

After adjustment, a higher frequency of tea intake was found to be negatively associated with the risk of hospitalized nephrolithiasis. Compared with participants who never drank tea, the odds ratio (95% confidence interval) [OR (95% CI)] for participants who drank ≥1 cup (180 mL) of tea per day was 0.418 (0.192–0.911) (*P* for trend = 0.013). Moreover, the adjusted OR (95% CI) for participants who drank ≥1 cup of green tea and black tea per day was 0.189 (0.069–0.520) (*P* for trend <0.001) and 1.248 (0.437–3.559) (*P* for trend = 0.654), respectively.

**Conclusions:**

Increased tea intake was found to be associated with a lower risk of hospitalized nephrolithiasis among Chinese adults. This finding may assist in the prevention of hospitalized nephrolithiasis.

## Introduction

Nephrolithiasis is one of the important public health problems because of its high prevalence and recurrence. The prevalence of kidney stones ranges from 3 to 14% worldwide, which has increased dramatically (1–3). Within 5 years of the initial episode, the recurrence rate becomes as high as 30% (4). Although most patients with nephrolithiasis are asymptomatic, the rate at which they suffer from a symptomatic episode or need surgical intervention is 10–25% per year and 48.5% in 5 years (5–7). Moreover, symptomatic nephrolithiasis may cause urinary tract obstruction, sepsis, renal insufficiency, and even death, which needs to be hospitalized (8, 9). Therefore, hospitalized nephrolithiasis requiring surgical intervention needs to be prevented.

The factors contributing to the increased burden of nephrolithiasis in the general population have not been well determined. Changing dietary habits is a key to preventing stone formation and recurrence (10). Tea is the most popularly consumed beverage worldwide (11). It is a source of caffeine that is thought to moderately increase diuretics. Diuretic effects can counteract high urinary calcium and reduce kidney stone formation (12). In addition, tea contains phytochemicals with strong antioxidant properties (13, 14). Many studies have shown that tea has a potential effect on stone formation (14–18). In a prospective population-based cohort comprising 502,621 participants, 12,407 had kidney stones. The findings of the cohort revealed that increased tea consumption was associated with a lower risk of kidney stones among Chinese adults after a median of 11.1 years of follow-up (19). In two prospective cohorts comprising 319,211 and 696,950 participants with 1,202 men and 1,451 women reported kidney stones, green tea intake was found to be associated with a lower risk of kidney stones among Chinese adults, compared with participants who never drank tea. The hazard ratio (HR) (95% CI) for participants who consumed ≥150 g/month dried tea leaves was 0.87 (0.70–1.08) for women and 0.67 (0.56–0.80) for men. However, the diagnosis of nephrolithiasis in previous studies was either self-reported or done at a physical examination center, which might be asymptomatic and did not require surgical intervention. Hospitalized nephrolithiasis is more of a concern due its severe complications. Compared with nephrolithiasis diagnosed in prior studies, hospitalized nephrolithiasis is more severe, likely to cause end-stage kidney disease because of obstructive nephropathy and even lead to death, which brings terrible medical burden (20). Therefore, the diagnosis of nephrolithiasis in prior studies may lead to bias of results. Moreover, a low urine volume caused by insufficient fluid consumption or excessive fluid loss is one of the most crucial risk factors for kidney stone formation (21). However, in previous studies, fluid intake was rarely adjusted, especially water consumption, resulting in unreliable association between tea intake and kidney stone formation. Besides, the subgroup analysis according to the types of tea was not performed in most previous studies. Tea is a popular drink in China. Therefore, this case–control study aimed to determine the effect of the intake of different types of tea on the risk of hospitalized nephrolithiasis after adjustments of fluid intake in Chinese adults.

## Materials and methods

### Participants

**FIGURE 1:**
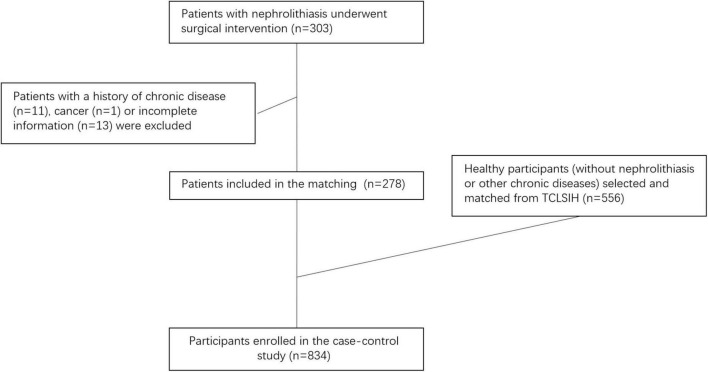
Flowchart.

The present study was conducted based on the Tianjin Chronic Low-Grade Systemic Inflammation and Health (TCLSIH) Cohort Study, a large, prospective dynamic cohort study which launched in Tianjin, China since 2007 (22). The design and data collection of the TCLSIH has been described in detail in a previous study (23). Since 2019, sub-cohorts of the TCLSIH have been launched in 9 cities, including Shenyang, across China. All the hospitalized nephrolithiasis cases and the healthy controls in the present study were selected from the Shenyang sub-cohort of the TCLSIH. A total of 303 patients with hospitalized nephrolithiasis in the Department of Urology at Shengjing Hospital of China Medical University from December 2019 to January 2021 were recruited for this study. All patients underwent surgical intervention. Patients with incomplete information or chronic disease (cancer, cardiovascular disease, or type 2 diabetes) (*n* = 25) were excluded from this study. Finally, 278 patients with hospitalized nephrolithiasis were included for analysis. Further, 556 healthy participants without nephrolithiasis or other chronic diseases (cancer, cardiovascular disease, or type 2 diabetes) were selected and matched by age (±1 year) and sex using the 1:2 ratio (Figure 1). Finally, a total of 834 participants (278 hospitalized nephrolithiasis patients and 556 healthy controls) were enrolled in the present case-control study.

The protocol of this study was approved by the ethics committee of the Shengjing Hospital of China Medical University (2019PS679K) and performed following the 1974 Declaration of Helsinki. All participants gave written informed consent.

### Assessment of dietary data

The information on the usual intake of foods and beverages (including different types of tea intake) was assessed using a 100-item validated semiquantitative food frequency questionnaire (FFQ) with specified serving sizes (24). The FFQ was designed to measure the food and beverage intake of the participants a month in the previous months. The FFQ represented the long-term food and beverage intake of the participants because the reproducibility and validity of the questionnaire were assessed. The response in the FFQ for food intake included a range of seven frequencies from *almost never* to *two or more times per day*. The response to questions related to beverage intake included eight frequencies ranging from *almost never drank* to ≥ *times per day*. The reproducibility and validity of the FFQ were obtained by comparing the data from the questionnaire with those from two dietary questionnaires collected approximately 3 months apart and weighed dietary records (WDRs) in 150 randomly selected participants from the TCLSIH cohort. The Spearman’s rank correlation coefficient for energy intake between the two FFQs administered 3 months apart was 0.68. The correlation coefficients for food items (fruits, vegetables, fish, meat, and beverages) between the two FFQs administered 3 months apart ranged from 0.62 to 0.79. The Spearman’s rank correlation coefficient for energy intake by the WDRs and the FFQ was 0.49. The correlation coefficients for nutrients (quercetin, vitamin C, vitamin E, polyunsaturated fats, saturated fats, carbohydrates, and calcium) between the WDRs and the FFQ ranged from 0.35 to 0.54. The daily intake of nutrients was calculated using an *ad hoc* computer program developed to analyze the questionnaire. The intake of food items was calculated from portion size (g/time) and the frequency of each food item consumed per day (25, 26). Each daily food intake was calculated by multiplying the frequency of consumption by the portion size of each food. The Chinese food composition tables were used as the nutrient database (27). By combining the information from the FFQ with the Chinese food composition table, the total energy intake was calculated by first multiplying the amount of consumption for each food item by its nutrient content and then summing nutrient contributions across all food items.

### Assessment of hospitalized nephrolithiasis

The diagnosis of nephrolithiasis was based on surgery. All patients with hospitalized nephrolithiasis underwent surgical intervention, including percutaneous nephrolithotomy, shock wave lithotripsy, ureteroscopy for renal stones, and so on. Patients with nephrolithiasis were identified using International Classification of Diseases-10 (ICD-10) codes N20 (as either a primary or a secondary diagnosis).

### Assessment of covariant variables

Detailed information was collected via a questionnaire for each participant. Anthropometric parameters (height and weight) were recorded using a standard protocol. The body mass index (BMI) was calculated as weight in kilograms divided by height in squared meters. The sociodemographic variables included sex, age, education, household income, smoking behavior (smoker, former smoker, never smoker), drinking behavior (drinker, former drinker, never drink), self-reported diagnoses of other chronic diseases, and water drinking per day according to 250 mL per cup. The education levels of the participants were divided into three categories: below college graduate, college graduate, or higher than college graduate.

### Statistical analysis

Descriptive data were presented as least-square means and 95% confidence interval (CI) or as percentages and examined using the analysis of covariance or chi-square test to characterize participants according to categories of tea drinking. The participants were divided into three groups based on the frequency of tea intake (never, <1 cup per day, or ≥1 cup per day). The associations between categories of tea intake and nephrolithiasis status were examined using conditional logistic regression analysis. The odds ratio (OR) and 95% CI were calculated. The linear trend across groups of tea intake was calculated by coding the categories of tea drinking as a continuous variable (never: 1; <1 cup per day: 2; ≥1 cup per day: 3) based on conditional logistic regression. Model 1 was used to calculate the crude OR; model 2 was adjusted for BMI; and model 3 was additionally adjusted for water intake per day, household income, educational level, smoking status, drinking status, and total energy, protein, and fat intake per day. Subgroup analyses according to the type of tea (green tea or black tea) were also performed. All analyses were performed using the Statistical Analysis System 9.3 edition for Windows (SAS Institute Inc.). All *P* values were two-tailed, and the difference was defined as significant when *P* < 0.05.

## Results

The characteristics of participants according to frequencies of tea intake (never, <1 cup per day, or ≥1 cup per day) are presented in Table 1. Compared with participants with lower intake of tea, those with higher consumption of tea tended to be men, current smokers, those having a high level of education and household income, and those having a greater intake of energy, proteins, fats, and vegetables, but lower intake of carbohydrates.

**TABLE 1:** Characteristics of participants by frequency of dietary tea intake.

	All participants	Frequency of dietary tea intake			*P* for trend^a^
		never	<1 cup per day	≥1 cup per day	
Participants (*n*)	834	352	267	215	–
Participants with nephrolithiasis (*n*, %)	278 (33.3)	178 (50.57)	58 (21.72)	42 (19.53)	–
Age (y)	53.87 (53.01–54.74)^b^	54.77 (53.44–56.10)	52.33 (50.80–53.85)	54.30 (52.60–56.00)	0.052
Sex (males, *n*, %)	557 (66.79)	203 (57.67)	180 (67.42)	174 (80.93)	<0.001
BMI	24.90 (23.96–25.49)	24.47 (24.09–24.84)	25.30 (24.87–25.73)	25.41 (24.93–25.88)	0.002
Total energy intake (kcal/d)	1621 (1574.12–1669.28)	1442.66 (1372.22–1513.10)	1610.88 (1530.01–1691.76)	1928.25 (1838.13–2018.38)	<0.001
Water intake^c^ (%)	41.95	38.46	43.40	46.39	0.221
Fat intake (g/d)	42.02 (41.37–42.67)	40.86 (39.85–41.87)	42.91 (41.76–44.06)	42.82 (41.51–44.14)	0.002
Carbohydrate intake (g/d)	239.81 (237.41–242.20)	246.37 (242.68–250.06)	237.62 (233.45–241.80)	231.77 (227.00–236.55)	<0.001
Meat (g/d)	55.76 (52.75–58.76)	53.94 (49.25–58.63)	58.23 (52.92–63.54)	55.65 (49.58–61.72)	0.668
Vegetable (g/d)	202.91 (195.86–209.96)	192.27 (181.33–203.21)	200.04 (187.66–212.42)	223.90 (209.74–238.05)	0.007
Bean (g/d)	42.31 (40.05–44.58)	41.88 (38.34–45.43)	41.80 (37.79–45.81)	43.65 (39.07–48.24)	0.556
Sodas (ml/d)	27.97 (23.33–32.62)	21.89 (14.64–29.14)	30.70 (22.50–38.90)	34.55 (25.17–43.93)	0.040
Income (>10,000 Yuan, %)	23.82	16.77	30.42	26.79	<0.001
Education level (college graduate or higher, %)	33.66	21.97	43.40	40.57	<0.001
**Smoke status (%)**					
Smoker	29.20	22.46	32.14	36.59	0.001
Former smoker	10.37	6.59	10.71	16.10	0.002
Never smoke	60.43	70.96	57.14	47.32	<0.001
**Drink status (%)**					
Drinker	14.60	16.29	10.90	16.43	0.117
Former drinker	11.58	11.71	10.15	13.15	0.593
Never drink	37.27	49.71	33.08	22.07	<0.001

BMI, body mass index.

^a^Analysis of covariance or chi-square test.

^b^Mean (95% confidence interval) (nutrients and foods intake were presented as least square means, adjusted by energy intake per day). ^c^Daily water intake ≥6 cups, according to 250 mL per cup.

Table 2 shows the crude and multivariable-adjusted associations between tea intake (subgrouped by green tea and black tea) and hospitalized nephrolithiasis. In the crude model, higher tea consumption was associated with a lower risk of hospitalized nephrolithiasis. After adjusting the BMI, the associations weakened. In the final multivariate model, compared with participants who never drank tea, the adjusted OR (95% CI) of hospitalized patients with nephrolithiasis who drank <1 cup per day and ≥1 cup per day of tea was 0.423 (0.216–0.830) (*P* for trend <0.001) and 0.418 (0.192–0.911) (*P* for trend = 0.013), respectively. In the subgroup analyses, higher consumption of green tea and black tea was associated with a decreased risk of nephrolithiasis in the crude model and model 2. However, after multivariable adjustments, only green tea intake was negatively associated with the risk of nephrolithiasis. Compared with participants who never drank green tea or black tea, the OR (95% CI) of hospitalized patients with nephrolithiasis who drank ≥1 cup per day of green and black tea was 0.189 (0.069–0.520) (*P* for trend < 0.001) and 1.248 (0.437–3.559) (*P* for trend = 0.654), respectively.

**TABLE 2:** Adjusted association between tea intake and nephrolithiasis.

	Frequency of dietary tea intake			*P* for trend^a^
	Never	<1 cup per day	≥1 cup per day	
**All tea**				
Participants (*n*)	352	267	215	
Participants with nephrolithiasis (*n*)	178	58	42	
Crude model	1.00 (reference)	0.299 (0.209–0.438)^b^	0.248 (0.165–0.373)	<0.001
Model 2*	1.00 (reference)	0.315 (0.218–0.455)	0.262 (0.172–0.397)	<0.001
Model 3**	1.00 (reference)	0.423 (0.216–0.830)	0.418 (0.192–0.911)	0.013
**Green tea**				
Participants (*n*)	408	288	138	
Participants with nephrolithiasis (*n*)	200	58	20	
Crude model	1.00 (reference)	0.259 (0.179–0.374)	0.166 (0.097–0.283)	<0.001
Model 2*	1.00 (reference)	0.262 (0.179–0.383)	0.175 (0.102–0.300)	<0.001
Model 3**	1.00 (reference)	0.320 (0.154–0.663)	0.189 (0.069–0.520)	<0.001
**Black tea**				
Participants (*n*)	513	237	84	
Participants with nephrolithiasis (*n*)	202	48	28	
Crude model	1.00 (reference)	0.412 (0.288–0.589)	0.799 (0.487–1.311)	<0.001
Model 2*	1.00 (reference)	0.425 (0.294–0.617)	0.846 (0.509–1.405)	0.004
Model 3**	1.00 (reference)	1.087 (0.559–2.114)	1.248 (0.437–3.559)	0.654

^a^Multivariate conditional logistic regression.

^b^Odds ratio (95% confidence interval) (all such values).

*Adjusted for BMI.

**Additionally adjusted total energy intake, water intake, protein intake, fat intake, carbohydrate intake, meat, vegetable, bean, sodas, education level, household income, smoking status and drinking status based on Model 2.

## Discussion

Nephrolithiasis is one of the most common urological disorders, which leads to high medical costs and social burdens. Severe nephrolithiasis requires surgical treatment and hospitalization, which may cause septic shock, renal insufficiency, and even death (8, 9). Tea (*Camellia sinensis* (L.) O. Kuntze) is one of the most popular drinks worldwide. The consumption of green tea is greater in northern China (11). In recent years, tea consumption in China has increased. Thus, assessing the association between tea intake and nephrolithiasis is meaningful. Fluid intake, especially water consumption, as a crucial factor for kidney stone formation, was not adjusted in previous studies. Therefore, this study aimed to explore the effects of tea intake on nephrolithiasis after adjusting for water intake. In the present case–control study, tea intake was negatively associated with the risk of hospitalized nephrolithiasis in Chinese adults independent of sociodemographic, behavioral, metabolic, and dietary factors (including water intake). Moreover, in the subgroup analyses, the negative association between tea intake and the risk of hospitalized nephrolithiasis was only found in green tea, but not in black tea.

In line with the results of this study, a previous population-based prospective cohort study with 439,072 participants (2,057 participants with kidney stones) found that higher consumption of tea was associated with a reduced risk of kidney stones, compared with non-tea consumers. The HR for participants who drank 200 mL/day was 0.95 (0.92–0.99) (18). A study based on another ongoing cohort involving 194,095 participants reported a declined risk of kidney stones among participants who drank one glass of tea per day compared with those drinking <1 glass per week (HR = 0.89, 95% CI: 0.82–0.97) (14). These studies were performed in Caucasian population. A limitation of these cohort study is the association between tea and nephrolithiasis subgrouped by the categories of tea did not be further explored. Our study made distinction between green and black tea and found that only green tea had a potential protective effect on kidney stone formation but not black tea. The Shanghai Men’s and Women’s Health Study found that green tea intake was associated with a lower risk of kidney stones among Chinese adults, compared with never drinkers; the HR (95% CI) for participants who consumed ≥ 150 g/month dried tea leaves was 0.87 (0.70–1.08) for women and 0.67 (0.56–0.80) for men (28). The results of a prospective population-based cohort that recruited 502,621 Chinese participants indicated that increased tea consumption was associated with a lower risk of kidney stones compared with non-tea consumers. The HR (95% CI) for participants who drank ≥7 cups of tea per day was 0.73 (0.65–0.83) (19). The results of these studies performed in Chinese population were consistent with our findings. However, neither of the aforementioned studies adjusted for water consumption, subsequently leading to unreliable results. The diagnosis of nephrolithiasis in prior studies was either self-reported or detected by a medical center, resulting in a non-differential misclassification of disease status, driving associations toward the null. Previous studies addressing in this issue had opposite conclusions. a prospective study enrolled 215 patients and 215 controls found that high consumption of tea increased risk of calcium oxalate kidney stone formation among general adults. Compared with participants who drank <2 glasses of tea/d, the OR (95% CI) for participant who drank ≥4 glasses were 2.73 (1.50–4.99) (29). This risk may be attributable to black tea though without assessment and higher carbohydrate intake which increased the urinary calcium excretion in study population. Another limitation is that the consumption of tea is highly estimated.

Catechin is an important component of polyphenols in tea and has an antioxidant function. Many studies suggested that the protective effect of green tea on nephrolithiasis was based on the presence of catechins (30, 31). Green tea retained the most catechins without fermentation, while black tea lost more than 75% catechins during fermentation (32). The present study hypothesized that this was the reason why high consumption of black tea could not decrease the risk of nephrolithiasis. Tea is a source of caffeine, which has been considered to moderately increase diuretics (33). Many studies suggested a protective effect of caffeine on kidney stone formation because the diuretic effect of caffeine counteracted hypercalciuria (16, 25). Urinary oxalate is an important risk factor for calcium oxalate stone formation (34). The oxalate content is about 0.8–14.0 mg/100 mL in the brewed green tea and 3.9–6.3 mg/100 mL in the brewed black tea (35, 36). However, data from a cohort study suggested only a modest positive association between dietary oxalate intake and the risk of kidney stone formation (37). Besides, the consumption of the oxalate-rich black tea did not significantly alter 24-h urinary oxalate excretion (38). The modest effects of urinary oxalate might have been offset by a large increase in fluid intake while drinking tea.

This study had several strengths. First, we investigated the association between total tea intake frequency and different types of tea and the risk of hospitalized nephrolithiasis. The evidence for the association between the intake of different types of tea and the risk of nephrolithiasis was very limited, and the present study extended the evidence in this regard (18, 19, 29). Second, the exact fluid intake was adjusted, which included water and sodas, to explore the association between tea intake and the risk of hospitalized nephrolithiasis. Third, the recruited patients had severe nephrolithiasis that required surgical treatment and hospitalization. However, the diagnosis of nephrolithiasis was self-reported by participants or detected by a medical center, in which surgical intervention might not be required.

This study also had several limitations. First, it was a case–control study, which made it impossible to infer causality. Second, recall bias, overreporting, and incorrect portion sizes were found during the questionnaire survey. The concentration for tea intake were not collected in present study. Third, the composition of stones was not analyzed for participants. Finally, residual or unmeasured confounding might still exist. Nevertheless, this study was novel in exploring the association between tea intake and hospitalized nephrolithiasis by adjusting extensive confounding factors, including fluid intake, based on Chinese population. This conclusion should further be confirmed through large-scale prospective studies in the future. An appropriate diet therapy can reduce the risk of urinary stone formation based on the potentially beneficial ingredients. The present study adds to the evidence that intake of green tea was associated with a lower risk of hospitalized nephrolithiasis. Encouraging increasing the green tea consumption is a priority for convenient and effective diet therapy to decrease the risk of hospitalized nephrolithiasis. Further studies are needed to investigate the causality and underlying mechanisms.

## Conclusion

The present study demonstrated that increased tea intake was significantly associated with a decreased risk of hospitalized nephrolithiasis among Chinese adults, which was attributed to green tea, but not black tea. This finding might assist in the prevention of hospitalized nephrolithiasis.

## Data availability statement

The original contributions presented in this study are included in the article/supplementary material, further inquiries can be directed to the corresponding author.

## Ethics statement

The studies involving human participants were reviewed and approved by the Ethics Committee of Shengjing Hospital Affiliated China Medical University (No. 2019PS679K). The clinical research registry UIN is ChiCTR1900028397. The patients/participants provided their written informed consent to participate in this study.

## Author contributions

SBa: full access to all the data in the study and takes responsibility for the integrity of the data and the accuracy of the data analysis; protocol/project development. YL, SBi, HL, JS, YX, KN, and SBa: data collection or management. YL, YX, and SBa: data analysis. YL and SBa: manuscript writing and editing. All authors contributed to the article and approved the submitted version.
